# Changes in tuberculosis burden and its associated risk factors in Guizhou Province of China during 2006–2020: an observational study

**DOI:** 10.1186/s12889-024-18023-w

**Published:** 2024-02-20

**Authors:** Yun Wang, Huijuan Chen, Xiaoqi Zeng, Long Liao, Xiaolong Lu, Aihua Zhang

**Affiliations:** 1 Key Laboratory of Environmental Pollution Monitoring and Disease Control, Ministry of Education, School of Public Health, Guizhou Medical University, Guiyang, Guizhou, China; 2 Department of Tuberculosis Prevention and Control, Guizhou Center for Disease Prevention and Control, Guiyang, Guizhou, China; 3 School of Medicine and Health Management, Guizhou Medical University, Guiyang, Guizhou, China

**Keywords:** Tuberculosis, Burden, Trend, Age-standardized rate, Guizhou

## Abstract

**Background:**

Understanding the trends of tuberculosis (TB) burden and its risk factors at the provincial level in the context of global End TB targets is crucial to identify the progress and challenges in TB control. We aimed to estimate the burden of TB and risk factors for death from 2006 to 2020 for the first time in Guizhou Province, China.

**Methods:**

Data were collected from the national TB surveillance system. Four indicators of TB burden and their corresponding age-standardized rates (ASRs), including incidence (ASIR), prevalence (ASPR), mortality (ASMR) and disability-adjusted life years (DALYs) (ASDR), were estimated and stratified by year, age, gender and prefecture. Temporal trends of ASRs were presented by locally weighted regression, and the annual percentage change was calculated. The correlation between gross domestic product (GDP) per capita and ASRs was evaluated by Pearson correlation analysis. The associated risk factors for death in PTB patients were determined using logistic regression models.

**Results:**

A total of 557,476 pulmonary TB (PTB) cases and 11,234 deaths were reported, including 2233 (19.9%) TB specific deaths and 9001 (80.1%) deaths from other causes. The 15-year average incidence, prevalence and mortality rates were 94.6, 102.6 and 2.1 per 100,000 population, respectively. The average DALY rate was 0.60 per 1000 population. The ASIR and ASPR have shown downward trends since 2012, with the largest percentage decrease in 2020 (ASIR: -29.8%; ASPR: -30.5%). The number in TB specific deaths consistently decreased during the study period (*P*<0.001), while the increase in deaths from other causes drove the overall upward trend in ASMR and ASDR. Four ASRs remained high in males and 5 prefectures. GDP per capita was negatively associated with the ASIR, ASPR and ASDR (*P*<0.05). Among PTB patients, men, patients with no fixed job, those with a low GDP level, patients with increasing age, those previously treated, those with severe symptoms, those transferred in and those receiving directly observed treatment were more likely to suffer death.

**Conclusion:**

Guizhou has made progress in reducing PTB cases and TB specific deaths over the last 15 years. Targeted interventions are needed to address these risk factors for death in PTB patients and high-risk areas.

## Background

Tuberculosis (TB) is an infectious disease caused by the bacillus *Mycobacterium tuberculosis* [1]. The sites typically affected by the disease are the lungs (pulmonary TB, PTB), but there are also other affected sites (extrapulmonary TB) [1]. TB was the leading cause of death as a single infectious agent worldwide before the pandemic of coronavirus disease 2019 (COVID-19) [1]. The End TB Strategy remains a priority for the World Health Organization (WHO) [2]. In recent years, encouraging progress in reducing TB burden has been made globally. However, the COVID-19 pandemic has reversed these gains [1]. Redoubling efforts and investments are therefore essential to mitigate the TB burden worldwide.

Estimating the trends of the TB burden is fundamental to guide effective control strategies for each country. China is one of the highest burden countries for TB in the world [2]. To fight this disease, China implemented three national plans for TB prevention and control between 2001 and 2020, covering three periods: period I (2001-2010) [3], period II (2011-2015) [4] and period III (2016-2020) [5]. During the three periods, China has been working to expand the directly observed treatment short-course strategy (DOTS) to the entire country [3, 6], strengthen the national surveillance systems to capture all PTB cases [7], improve the TB health-care system [4–6], and protect patients from the financial burden of TB treatment [5, 6]. Under the guidance of these national policies, China has made remarkable progress in TB control, but it is still far from achieving the End TB targets [5, 6]. Thus, understanding China’s TB status is urgently needed, especially at the provincial level. Knowledge of which province lags behind in controlling TB can inform effective intervention efforts that aim to promote progress in achieving the End TB targets at the national level.

Guizhou is a province in southwestern China with a low economic level and the third highest TB incidence [8]. There are 9 prefectures and 88 counties with a population of 38.56 million [9]. In keeping with the national pace, Guizhou also implemented three provincial TB plans in three corresponding periods [8, 10, 11]. In 2005, a national TB Information Management System (TBIMS) was established based on the internet to monitor real-time and detailed information on PTB nationwide [7]. The platform has been fully implemented in Guizhou since 2006 to collect information on PTB patients. The available data from the TBIMS platform can be used to track the PTB burden. However, no study to date has assessed the changes in the TB burden in this province using data from this platform. An assessment of TB burden trends is crucial to identify the progress and challenges in TB control. Tracking the risk factors associated with disease burden can help develop effective control measures to appropriately address these issues.

The Global Burden of Diseases study (GBD) provides a systematic methodology to quantify the burden of disease and its risk factors [12]. This methodology has been widely used at the population-level to estimate TB [13–20] and other disease burdens [21–25] worldwide using data from the GBD database [13–20, 24, 25] or national surveillance system [21–23]. Four indicators are applied to measure the burden of disease. They are incidence [14, 15, 17–20, 24, 25], prevalence [13, 15, 17, 18, 20, 24], mortality [13, 15, 17–20, 24, 25], and disability-adjusted life years (DALYs) [13, 16, 17, 20–25]. However, only a few studies included all four metrics [17, 20, 24]. Comparison is at the heart of the disease burden approach [12]. Thus, each metric is generally examined over time and stratified by gender [13, 15, 16, 19–22, 24, 25], age [13, 15–19, 21–23, 25], geographic location [14–16, 19, 20, 22–25] and level of socioeconomic development [15, 19, 20, 25]. Reducing death in patients with TB is a concerted global target [2]. WHO defined death in TB patients as:“A TB patient who dies for any reason before starting or during the course of treatment” [26]. In China, the proportion of TB specific death among registered TB patients is an important indicator of patient care management, which provides key evidence for the evaluation of TB control effectiveness and quality [27]. Thus, TB specific death and death from other causes are reported separately in TBIMS database. TB specific death refers to TB disease complications-specific mortality among TB patients. Death from other causes is mortality among TB from other causes [28]. Some studies have explored risk factors for all-cause death among TB patients [29–31]. Others distinguished TB specific death from all-cause death and addressed the factors associated with them [32, 33].

Based on the GBD methodology and literature review above, this observational study analyzed at the population level, aims to provide a comprehensive assessment of the PTB burden and risk factors for death in Guizhou to advance evidence-informed prevention plans. We collected data from the TBIMS database over a 15-year period from 2006 to 2020 and analyzed the PTB burden with the incidence, prevalence, mortality and DALYs. They were stratified by gender, age, prefecture and annual gross domestic product (GDP) per capita. The risk factors associated with all-cause death and TB specific death were further estimated.

## Methods

### Data sources

The data of all notified PTB cases and the population between January 2006 and December 2020 in Guizhou were collected from the TBIMS database. Approval for use of the database was granted by the Department of Tuberculosis Prevention and Control of Guizhou Center for Disease Prevention and Control. The study included a total of 557,476 patients who were diagnosed and registered during the study period. The remaining 348 patients were excluded either their time of diagnosis or registration was outside the study period, and data on their treatment outcomes were missing. The patients’ data extracted from the database were age, gender, ethnic group, occupation, home address, date of diagnosis, date of registration, registered residence at the time of TB diagnosis, anti-TB drug history, classification of patients, severity of disease, types of case finding, directly observed treatment (DOT) status throughout the entire treatment and treatment outcomes (all-cause death and TB specific death). Data on annual GDP per capita were obtained from the yearbook of Guizhou Provincial Bureau of Statistics [9]. Fundamental geographic data were downloaded from the National Geomatics Center of China to make the prefecture-level ring map of Guizhou.

### Definition of PTB cases and types of case findings

PTB is defined as bacteriologically confirmed or clinically diagnosed TB with lesions in the lungs [7, 26], including patients with both pulmonary and extrapulmonary TB [7]. Based on a history of previous TB treatment, PTB patient cases are classified as new patients and previously treated patients. The former is defined as a patient who has never received anti-TB treatment or taken anti-TB drugs for less than 1 month. The latter is defined as a patient who has been treated for TB in the past for at least 1 month [26].

Types of case finding are classified as passive case finding (PCF), active case finding (ACF) and transfer in [34, 35]. PCF refers to the way in which individuals present to health facilities for TB diagnosis after they have developed suspicious symptoms of TB and are aware of their symptoms [35]. ACF is defined as a systematic screening and clinical evaluation of people at high risk of developing TB for early detection of patients. It includes a variety of approaches, such as contact screening of TB patients at home or in the community [35]. Transfer refers to patients who were originally registered in other health facilities for TB care but transferred to the current health facilities to continue care [36].

### Statistical analysis

#### Descriptive analysis for general characteristics

Descriptive analysis was presented to understand the distribution of demographic characteristics of PTB cases in 3 periods (2006-2010, 2011-2015, 2016-2020). Frequencies and percentages were used for categorical variables, while medians and interquartile ranges (IQRs) were used for continuous variables. A ring map made by ArcGIS (version 10.4, ESRI Inc., Redlands, CA, USA) [37] was used to display the spatial–temporal patterns of PTB burden from 2006 to 2020 yearly at the prefecture level. Other statistical analyses in this study were performed by R (version 4.2.1, Vienna, Austria).

#### PTB burden analysis

According to the methodology of GBD online [12, 15, 19, 38], the number, rates and age-standardized rates (ASRs) of PTB incidence, prevalence, mortality, and DALYs were calculated and stratified by gender, age, geographic location and GDP per capita over time. Incident PTB cases were new patients, while prevalent PTB cases were all existing patients, including previously treated patients and new patients. Deceased PTB cases were recorded as patients who died for any reason during the study period. The rates and ASRs of incidence, prevalence, and mortality were expressed as the number of cases per 100,000 population [15, 19]. DALYs were presented in thousands [24].

ASR was calculated based on a global standard age structure [39], and its specific approaches have been described in detail elsewhere [20, 25]. The 95% uncertainty interval (UI) was assessed for corresponding rates (incidence, prevalence, mortality, and DALYs) and ASRs (age-standardized incidence rate, ASIR; age-standardized prevalence rate, ASPR; age-standardized mortality rate, ASMR; and age-standardized DALY rate, ASDR). The annual percentage change was calculated to show the changes in ASRs from 2006 to 2020 (percentage change = [(final value – starting value)/|starting value|] × 100).

The estimated annual percentage change (EAPC) was used to evaluate the temporal changes in ASRs over these 15 years. It was calculated as EAPC=100×[exp(β)−1]. β was obtained from a linear regression model: *Y* =*α*+*βX* +*ε,* where *Y* equals ln (ASR), *X* is the calendar year, and ε is the error term [20, 25]. The 95% confidence interval (CI) of the EAPC could also be obtained from the linear model [20]. When the EAPC and its 95% CI are positive, the ASR presents an upward trend; if both the EAPC and 95% CI are negative, the ASR is considered a downward trend; otherwise, the ASR is stable. Temporal trends of ASRs were smoothly presented by locally weighted regression (Loess) [40, 41].

#### The correlation between GDP per capita and PTB burden

Pearson correlation analysis was used to evaluate the correlation between GDP per capita and PTB burden (ASIR, ASPR, ASMR and ASDR) in 3 periods. Linear correlation diagrams with correlation coefficients (ɣ) and *P* values were used to present those results. The nine prefectures were ranked in descending order of average GDP per capita for each period.

#### Risk factors associated with all-cause death and TB specific death

Variables associated with all-cause death and TB specific death were explored initially by separate univariate analysis, and variables with a *P* value less than 0.2 were included in the separate multivariate logistic regression modeling process. The likelihood ratio test was assessed at each step and used to determine the final model where only variables with a* P* value ˂0.05 remained. The odds ratio (OR) and 95% CI were calculated for each predictive variable. A total of 10 variables were included in the analysis, including time periods, average GDP per capita over 15 years, gender, age, ethnic group, occupational status, classification of patients, severe symptoms status, types of case finding and DOT status.

### Ethical review

In this study, data were collected from routine TB surveillance system. The approval of the study were obtained from Guizhou Medical University, and the Department of Tuberculosis Prevention and Control of Guizhou Center for Disease Prevention and Control.

## Results

### Demographic characteristics of PTB patients

The demographic characteristics of all patients and their three subgroups are shown in Table 1. A total of 557 476 PTB cases were reported in Guizhou from 2006 to 2020. Three subgroups included the cases registered in 3 periods: subgroup 1 (from 2006 to 2010, *n*=167421), subgroup 2 (from 2011 to 2015, *n*=203013) and subgroup 3 (from 2016 to 2020, *n*=187042). Of all cases, 66.4% were male and 33.6% were female, with a 2.0 sex ratio. The median age was 42.0 (IQR 26.0~59.0) years. A total of 70.5% of cases were Han, and the cases were mainly farmers (77.8%), followed by migrant workers and unemployed individuals (11.2%). A total of 92.2% of patients were new cases, and 7.8% had previously received anti-TB therapy. Moreover, 48.3% of patients were found by PCF followed by transfer (33.0%). A total of 50.9% of patients received DOT during the whole treatment. In terms of the distribution of patients in 3 periods, the proportion of women, ethnic groups, patients aged 60 years and above, new patients and patients found by ACF gradually increased over time. However, the proportions of patients who received DOT and those found by PCF showed a downward trend.

**Table 1:** Characteristics of PTB cases in Guizhou Province from 2006 to 2020

Characteristic	Total	Three periods		
		2006~2010	2011~2015	2016~2020
Total n (%)	557476 (100.0)	167421(30.0)	203013(36.4)	187042(33.6)
Gender				
Female	187399 (33.6)	54435 (32.5)	67979 (33.5)	64985 (34.7)
Male	370077 (66.4)	112986 (67.5)	135034 (66.5)	122057 (65.3)
Age, Median (IQR)	42.0 (26.0, 59.0)	38.0(25.0, 56.0)	42.0(26.0, 59.0)	44.0(25.0, 61.0)
≤25	138970 (24.9)	41968 (25.1)	48714 (24.0)	48288 (25.8)
26-59	285846 (51.3)	92278 (55.1)	105283 (51.9)	88285 (47.2)
≥60	132660 (23.8)	33175 (19.8)	49016 (24.1)	50469 (27.0)
Ethnic group				
Han	393276 (70.5)	138452 (82.7)	135739 (66.9)	119085 (63.7)
Not Han	164200 (29.5)	28969 (17.3)	67274 (33.1)	67957 (36.3)
Occupation				
Fixed job workers and others	61555 (11.0)	16733 (10.0)	18939 (9.3)	25883 (13.8)
Migrant workers and unemployed	62190 (11.2)	22823 (13.6)	21270 (10.5)	18097 (9.7)
Farmers	433731 (77.8)	127865 (76.4)	162804 (80.2)	143062 (76.5)
Classification of patients				
New cases	513805 (92.2)	147814 (88.3)	189870(93.5)	176121(94.2)
Previously treated cases	43671 (7.8)	19607(11.7)	13143(6.5)	10921(5.8)
Types of case finding				
PCF	269144 (48.3)	94734 (56.6)	99851 (49.2)	74559 (39.9)
ACF	104318 (18.7)	20211 (12.1)	43271 (21.3)	40836 (21.8)
Transfer in	184014 (33.0)	52476 (31.3)	59891 (29.5)	71647 (38.3)
Underwent DOT during whole treatment				
Yes	284005 (50.9)	86965 (51.9)	105538 (52)	91502 (48.9)
No	273471 (49.1)	80456 (48.1)	97475 (48)	95540 (51.1)

*PTB* Pulmonary tuberculosis, *IQR* Interquartile ranges, *PCF* Passive case finding, *ACF* Active case finding, *DOT* Directly observed treatment

### Changes in PTB burden

#### Incidence of PTB

**Fig. 1:**
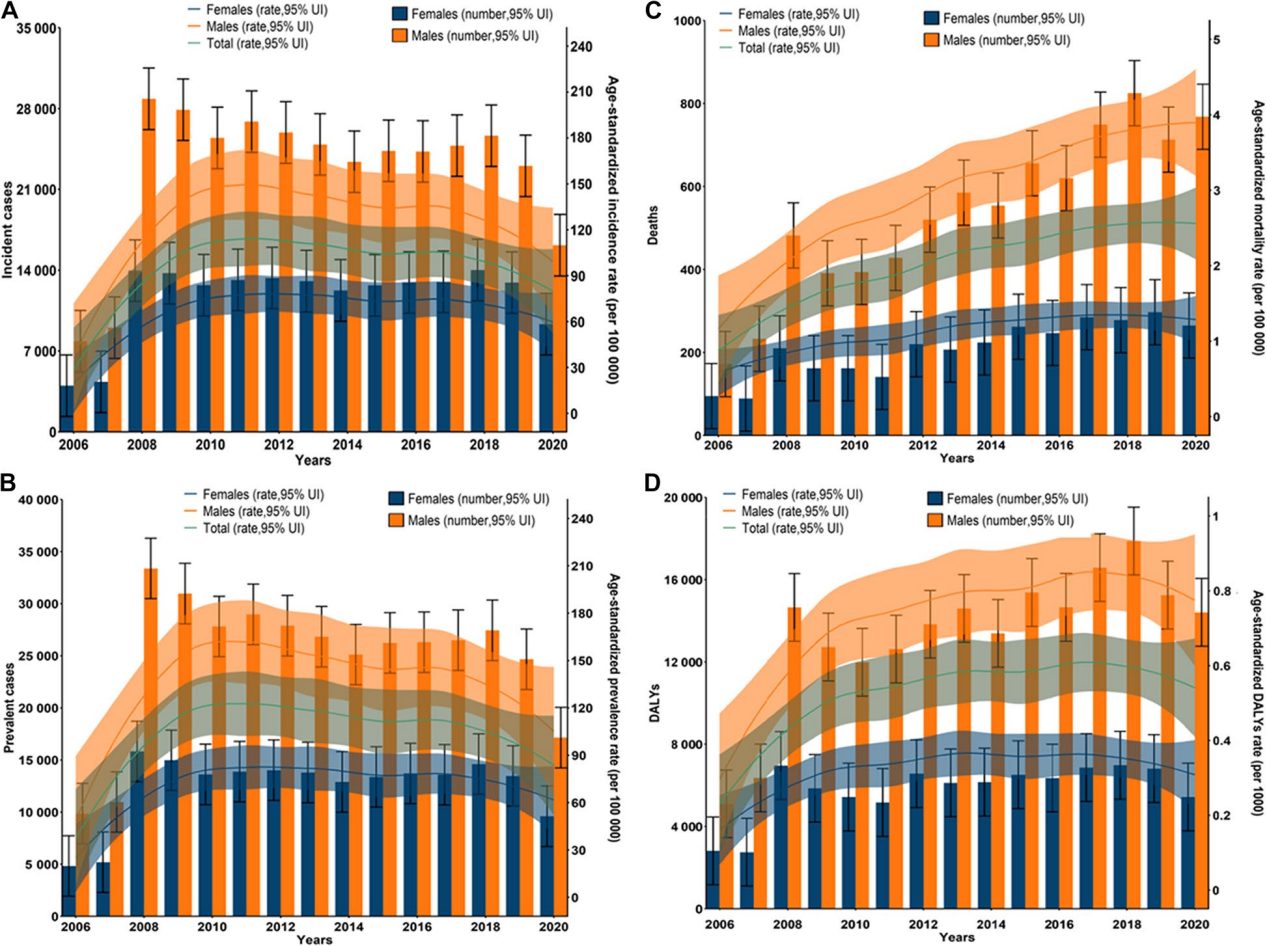
Trends in numbers and age-standardized rates of PTB burden in Guizhou Province, 2006-2020. **A** Trends of PTB incidence from 2006 to 2020. **B** Trends of PTB prevalence from 2006 to 2020. **C** Trends of PTB all-cause mortality from 2006 to 2020. **D** Trends of PTB DALYs from 2006 to 2020. Notes: PTB: pulmonary tuberculosis; DALYs: disability-adjusted life years

**Fig. 2:**
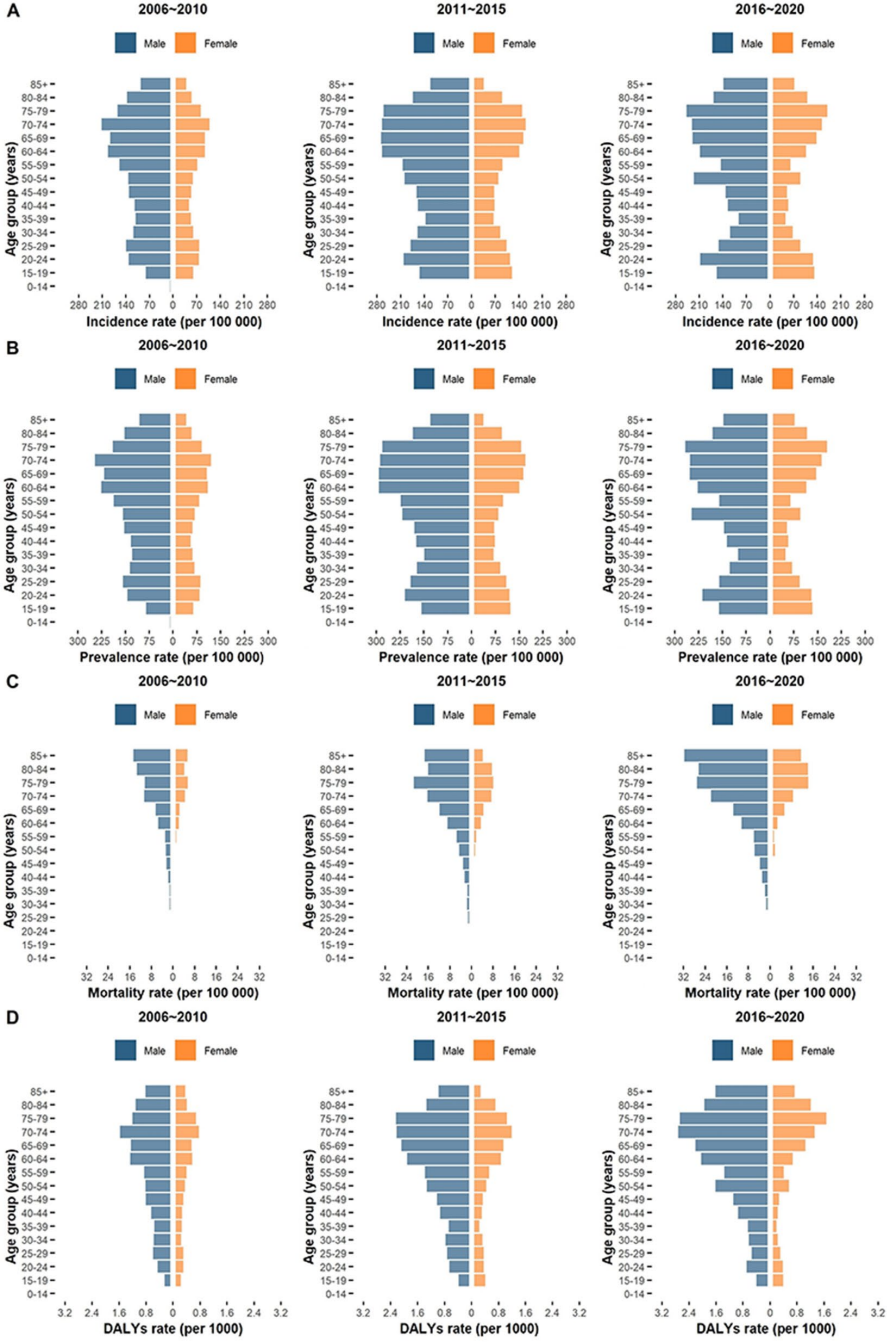
Distribution of PTB burden by gender and age in Guizhou Province, 2006-2020. **A** Distribution of PTB incidence rate in 3 periods. **B** Distribution of PTB prevalence rate in 3 periods. **C** Distribution of PTB all-cause mortality rate in 3 periods. **D** Distribution of PTB DALYs rate in 3 periods. Notes: PTB: pulmonary tuberculosis; DALYs: disability-adjusted life years

**Fig. 3:**
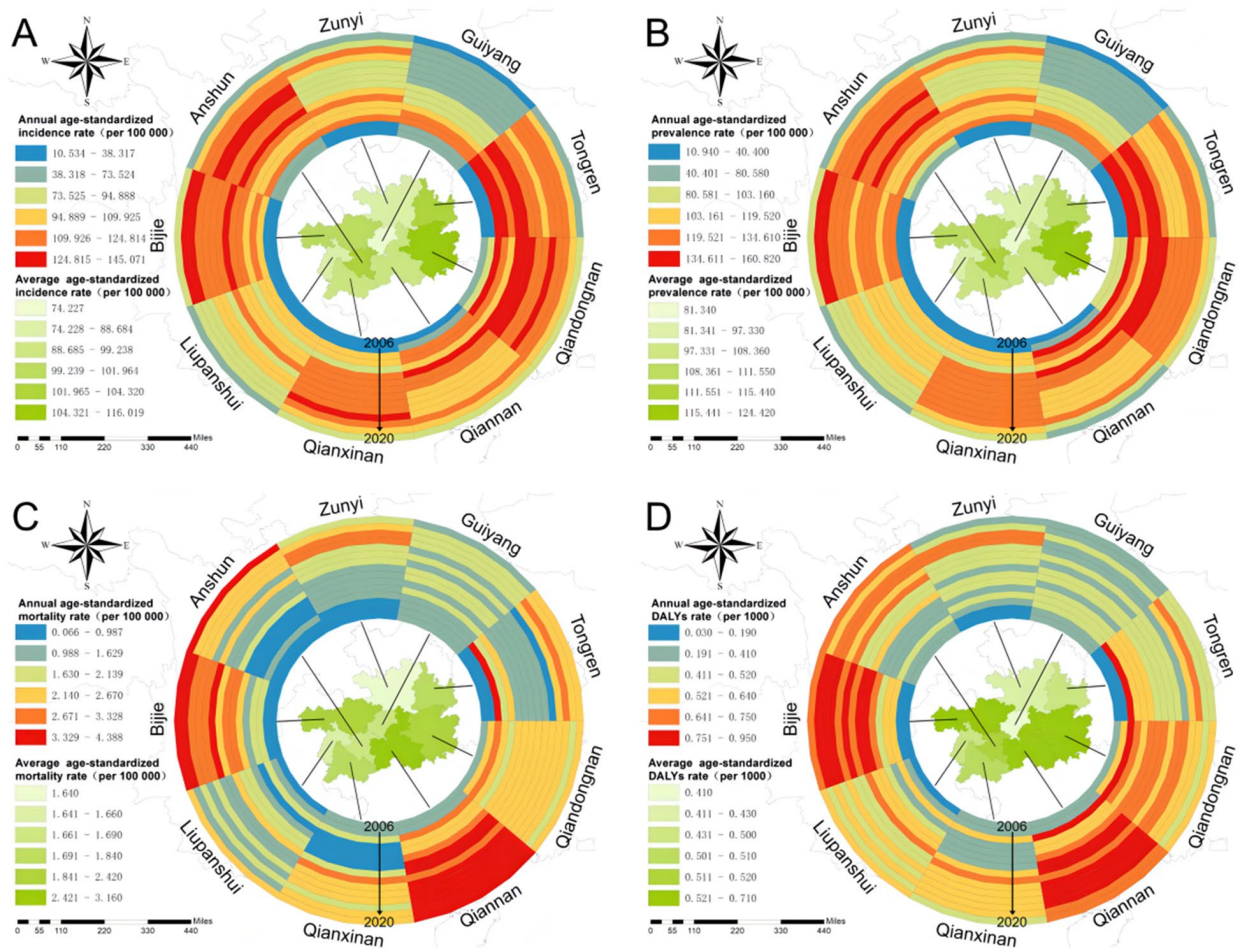
The ring map for spatial–temporal distribution of PTB burden in Guizhou Province, 2006-2020. **A** Spatial–temporal distribution of annual and average ASIR of PTB. **B** Spatial–temporal distribution of annual and average ASPR of PTB. **C** Spatial–temporal distribution of annual and average ASMR of PTB. **D** Spatial–temporal distribution of annual and average ASDR of PTB. Notes: PTB: pulmonary tuberculosis; ASIR: age-standardized incidence rate; ASPR: age-standardized prevalence rate; ASMR: age-standardized mortality rate; ASDR: age-standardized DALYs rate; DALYs: disability-adjusted life years

A total of 513 805 new PTB cases occurred in Guizhou from 2006 to 2020 (Table 2). The 15-year average incidence rate was 94.6 (95% UI: 82.2~107.0) per 100,000 population, with the highest ASIR in 2011 (118.6 [95% UI: 101.8~128.6]) (Table 2). During 2006 and 2010, the ASIR of PTB had a pronounced rising trend (EAPC=0.39, 95% CI 0.08~0.58) (Table 3). The upward trends in ASIR also occurred in both sexes (male: EAPC=0.40, 95% CI 0.09~0.58; female: EAPC=0.39, 95% CI 0.07~0.58) (Table 3, Fig. 1A). Among the 9 prefectures, the most obvious increasing trend was observed in Bijie (EAPC=0.96, 95% CI 0.20~1.14) (Table 3). During period II (2011-2015) and period III (2016-2020), the overall ASIR had a continuously decreasing trend (period II: EAPC=-0.04, 95% CI -0.05~-0.02; period III: EAPC=-0.08, 95% CI -0.18~0.00) (Table 3). Downward trends in ASIR were observed in both sexes (Fig. 1A). The most pronounced decline in ASIR from 2011 to 2020 was observed in 2020, with the highest percentage decrease of -29.8% (Table 2). For the distribution of incidence and ASIR by gender and age, all age groups were affected by PTB, and each group had more male than female cases (Figs. 1A and 2A). A high PTB incidence was observed in the middle- to old-age group (Fig. 2A). For geographic distribution, the ASIR of PTB remained high in 4 prefectures (Bijie, Anshun, Tongren and Qiandongnan) from 2006 to 2020 (Fig. 3A).

**Table 2:** Numbers, rates, age-standardized rates and percentage changes for PTB incidence, prevalence, mortality and DALYs from 2006 to 2020 in Guizhou

Year	**Incidence**				**Prevalence**				**Mortality**				**DALYs**			
	New cases No.	Rate per 100 000 No. (95% UI)	ASIR per 100 000 No. (95% UI)	Percentage change (%)	Prevalent cases No.	Rate per 100 000 No. (95% UI)	ASPR per 100 000 No. (95% UI)	Percentage change (%)	Died cases No.	Rate per 100 000 No. (95% UI)	ASMR per 100 000 No. (95% UI)	Percentage change (%)	DALYs No.	Rate per 1000 No. (95% UI)	ASDR per 1000 No. (95% UI)	Percentage change (%)
2006	11842	31.8(19.3~44.2)	32.4(18.3~45.2)	——	14680	39.4(26.5~52.3)	40.3(25.5~53.2)	——	267	0.7(0.4~1.1)	0.8(0.4~1.0)	——	7911	0.21(0.15~0.28)	0.22(0.16~0.28)	——
2007	13348	35.5(23.1~48.0)	36.3(22.1~48.9)	12.0	16161	43.0(30.1~56.0)	44.1(29.1~56.9)	9.4	322	0.9(0.5~1.2)	1.0(0.6~1.1)	25.0	9106	0.24(0.18~0.31)	0.26(0.20~0.32)	18.2
2008	42810	113.8(101.3~126.2)	115.5(100.4~127.2)	218.2	49203	130.8(117.9~143.7)	133.2(116.9~144.7)	202.0	692	1.8(1.5~2.2)	2.0(1.6~2.1)	90.9	21607	0.57(0.51~0.64)	0.60(0.54~0.66)	106.3
2009	41648	109.9(97.5~122.4)	110.8(96.5~123.3)	-4.1	45946	121.3(108.4~134.2)	122.5(107.4~135.2)	-8.0	553	1.5(1.1~1.8)	1.6(1.2~1.7)	-20.0	18576	0.49(0.43~0.55)	0.51(0.45~0.57)	-15.0
2010	38166	96.6(84.1~109.0)	97.5(83.2~110.0)	-12.0	41431	104.8(91.9~117.7)	106.0(90.9~118.7)	-13.5	556	1.4(1.1~1.8)	1.6(1.1~1.7)	0.0	17411	0.44(0.38~0.50)	0.46(0.40~0.52)	-9.8
2011	40025	115.2(102.8~127.6)	118.6(101.8~128.6)	21.6	42857	123.3(110.5~136.2)	127.2(109.5~137.2)	20.0	569	1.6(1.3~2.0)	1.7(1.4~1.9)	6.2	17788	0.51(0.45~0.58)	0.54(0.47~0.60)	17.4
2012	39241	113.1(100.7~125.6)	115.7(99.7~126.5)	-2.4	41905	120.8(107.9~133.7)	123.4(106.9~134.7)	-3.0	740	2.1(1.8~2.5)	2.1(1.8~2.4)	23.5	20396	0.59(0.52~0.65)	0.59(0.53~0.66)	9.3
2013	37936	108.9(96.4~121.3)	111.1(95.5~122.3)	-4.0	40629	116.6(103.7~129.5)	118.9(102.7~130.5)	-3.6	792	2.3(1.9~2.6)	2.3(2.0~2.6)	9.5	20717	0.59(0.53~0.66)	0.60(0.54~0.66)	1.7
2014	35641	101.8(89.3~114.2)	102.4(88.4~115.2)	-7.8	38011	108.5(95.6~121.4)	109.0(94.6~122.4)	-8.3	778	2.2(1.9~2.6)	2.1(1.9~2.5)	-8.7	19541	0.56(0.49~0.62)	0.55(0.49~0.61)	-8.3
2015	37027	105.6(93.1~118.0)	104.9(92.1~119.0)	2.4	39611	112.9(100.0~125.8)	112.0(99.0~126.8)	2.8	918	2.6(2.3~3.0)	2.5(2.3~2.9)	19	21890	0.62(0.56~0.69)	0.60(0.54~0.67)	9.1
2016	37224	105.5(93.0~117.9)	104.3(92.1~118.9)	-0.6	40013	113.4(100.5~126.3)	111.9(99.5~127.3)	-0.1	867	2.5(2.1~2.8)	2.2(2.2~2.7)	-12	21005	0.60(0.53~0.66)	0.56(0.50~0.63)	-6.7
2017	37780	106.2(93.8~118.7)	105.6(92.9~119.7)	1.2	40089	112.8(99.9~125.7)	111.8(98.9~126.7)	-0.1	1034	2.9(2.6~3.3)	2.6(2.6~3.2)	18.2	23444	0.66(0.60~0.72)	0.63(0.57~0.69)	12.5
2018	39667	110.8(98.4~123.3)	110.4(97.4~124.2)	4.5	42053	117.5(104.6~130.4)	116.9(103.6~131.4)	4.6	1103	3.1(2.7~3.4)	2.8(2.8~3.4)	7.7	24848	0.69(0.63~0.76)	0.66(0.60~0.72)	4.8
2019	35961	99.8(87.5~112.3)	99.6(86.5~113.3)	-9.8	38136	105.9(93.0~118.8)	105.3(92.0~119.8)	-9.9	1010	2.8(2.5~3.2)	2.5(2.5~3.1)	-10.7	22059	0.61(0.55~0.68)	0.58(0.52~0.64)	-12.1
2020	25489	70.4(57.9~82.8)	69.9(57.0~83.8)	-29.8	26751	73.8(61.0~86.7)	73.2(59.9~87.7)	-30.5	1033	2.9(2.5~3.2)	2.5(2.6~3.1)	0	19838	0.55(0.48~0.61)	0.51(0.45~0.57)	-12.1
Total	513805	94.6(82.2~107.0)			557476	102.6(89.7~115.5)			11234	2.1(1.7~2.4)			286137	0.60(0.54~0.66)		

*PTB* Pulmonary tuberculosis, *DALYs* Disability-adjusted life years, *ASIR* Age-standardized incidence rate, *ASPR* Age-standardized prevalence rate, *ASMR* Age-standardized mortality rate, *ASDR* Age-standardized DALYs rate, *UI* Uncertainty interval

**Table 3:** EAPCs of age-standardized rates for PTB incidence, prevalence, mortality and DALYs in three periods in Guizhou

**Characteristics**	**EAPC of ASIR No. (95% CI)**			**EAPC of ASPR No. (95% CI)**			**EAPC of ASMR No. (95% CI)**			**EAPC of ASDR No. (95% CI)**		
	**2006-2010**	**2011-2015**	**2016-2020**	**2006-2010**	**2011-2015**	**2016-2020**	**2006-2010**	**2011-2015**	**2016-2020**	**2006-2010**	**2011-2015**	**2016-2020**
**Overall**	0.39(0.08~0.58)	-0.04(-0.05~-0.02)	-0.08(-0.18~0.00)	0.34(0.05~0.54)	-0.04(-0.06~-0.02)	-0.08(-0.18~0.00)	0.21(0.01~0.37)	0.07(0.02~0.11)	0.02(-0.04~0.08)	-0.19(-0.41~-0.01)	-0.02(-0.05~0.02)	0.03(-0.04~0.09)
**Sex**												
Male	0.40(0.09~0.58)	-0.04(-0.06~-0.02)	-0.09(-0.18~0.00)	0.35(0.06~0.54)	-0.04(-0.06~-0.02)	-0.09(-0.19~-0.01)	0.23(0.02~0.39)	0.06(0.02~0.10)	0.04(-0.03~0.10)	-0.2(-0.42~-0.03)	-0.02(-0.05~0.02)	0.02(-0.05~0.08)
Female	0.39(0.07~0.58)	-0.03(-0.05~-0.01)	-0.07(-0.16~0.02)	0.34(0.05~0.54)	-0.03(-0.05~-0.01)	-0.07(-0.17~0.01)	0.17(-0.02~0.34)	0.09(0.00~0.17)	0.00(-0.05~0.05)	-0.17(-0.39~0.02)	-0.02(-0.07~0.04)	0.05(-0.02~0.11)
**Region**												
Bijie	0.96(0.20~1.14)	-0.01(-0.04~0.02)	-0.04(-0.15~0.07)	0.97(0.19~1.16)	-0.01(-0.05~0.02)	-0.05(-0.15~0.05)	1.19(0.10~1.46)	0.09(0.02~0.16)	0.06(0.01~0.11)	-0.52(-1.29~-0.17)	-0.04(-0.11~0.02)	-0.01(-0.07~0.05)
Tongren	0.76(0.17~0.96)	-0.06(-0.08~-0.05)	-0.10(-0.19~-0.02)	0.66(0.11~0.90)	-0.06(-0.08~-0.05)	-0.10(-0.20~0.00)	0.43(-0.04~0.76)	-0.03(-0.10~0.05)	0.21(-0.05~0.43)	-0.32(-0.74~-0.03)	0.06(0.02~0.10)	-0.04(-0.20~0.11)
Liupanshui	0.67(0.16~0.86)	-0.02(-0.06~0.03)	-0.11(-0.20~-0.05)	0.62(0.15~0.82)	-0.02(-0.07~0.04)	-0.13(-0.20~-0.07)	0.09(-0.17~0.33)	-0.13(-0.28~0.00)	0.05(-0.08~0.18)	-0.18(-0.40~0.00)	0.09(-0.01~0.19)	0.02(-0.06~0.11)
Zunyi	0.54(0.10~0.77)	-0.07(-0.10~-0.05)	-0.07(-0.20~0.05)	0.43(0.05~0.66)	-0.07(-0.10~-0.05)	-0.08(-0.21~0.04)	0.28(0.10~0.40)	0.10(0.05~0.14)	0.00(-0.15~0.14)	-0.23(-0.47~-0.04)	-0.02(-0.05~0.01)	0.04(-0.11~0.19)
Qianxinan	0.45(0.10~0.65)	0.02(0.00~0.04)	-0.09(-0.17~-0.02)	0.42(0.08~0.61)	0.01(-0.01~0.04)	-0.09(-0.17~-0.02)	-0.06(-0.29~0.15)	0.38(0.13~0.51)	-0.01(-0.07~0.04)	-0.05(-0.23~0.13)	-0.14(-0.26~-0.04)	0.05(0.01~0.09)
Qiannan	0.37(0.09~0.54)	-0.07(-0.09~-0.05)	-0.07(-0.15~0.01)	0.34(0.05~0.53)	-0.06(-0.09~-0.04)	-0.07(-0.16~0.01)	0.21(-0.02~0.40)	0.00(-0.12~0.13)	-0.01(-0.06~0.05)	-0.18(-0.42~0.03)	0.01(-0.06~0.09)	0.04(-0.02~0.10)
Anshun	0.21(0.04~0.34)	0.02(-0.02~0.07)	-0.18(-0.33~-0.06)	0.13(0.00~0.24)	0.02(-0.01~0.05)	-0.18(-0.33~-0.06)	-0.03(-0.23~0.17)	0.34(0.06~0.53)	0.21(0.07~0.31)	-0.03(-0.18~0.11)	-0.12(-0.24~-0.02)	-0.05(-0.14~0.03)
Guiyang	0.19(0.03~0.32)	-0.07(-0.12~-0.02)	-0.13(-0.22~-0.05)	0.16(0.00~0.29)	-0.06(-0.11~-0.02)	-0.13(-0.23~-0.05)	0.12(0.01~0.22)	0.06(-0.04~0.16)	-0.02(-0.15~0.11)	-0.11(-0.21~0.03)	0.01(-0.07~0.09)	0.07(-0.02~0.16)
Qiandongnan	0.11(-0.01~0.22)	-0.04(-0.06~-0.02)	-0.07(-0.11~-0.04)	0.09(-0.03~0.21)	-0.05(-0.07~-0.03)	-0.07(-0.11~-0.04)	0.10(-0.11~0.30)	0.01(-0.03~0.04)	-0.02(-0.11~0.07)	-0.1(-0.27~0.07)	0.02(-0.03~0.07)	0.06(0.02~0.10)

*EAPCs* Estimated annual percentage changes, *PTB* Pulmonary tuberculosis, *DALYs* Disability-adjusted life years, *ASIR* Age-standardized incidence rate, *ASPR* Age-standardized prevalence rate, *ASMR* Age-standardized mortality rate, *ASDR* Age-standardized DALYs rate, *CI* Confidence intervals

#### Prevalence of PTB

Over these 15 years, 557 476 prevalent PTB cases occurred in Guizhou (Table 2). The average prevalence rate was 102.6 (95% UI: 89.7~115.5) per 100,000 population, with the highest ASPR in 2008 (133.2 [95% UI: 116.9~144.7]) (Table 2). From 2006 to 2010, the ASPR had an upward trend, with an EAPC of 0.34 (95% CI 0.05~0.54) (Table 3). The trends of ASPR increased in both sexes (male: EAPC=0.35 [95% CI 0.06~0.54]; female: EAPC=0.34 [95% CI 0.05~0.54]) (Table 3). Among the 9 prefectures, the largest increasing trend occurred in Bijie (EAPC= 0.97, 95% CI 0.19~1.16) (Table 3). From period II (2011-2015) to period III (2016-2020), the overall ASPR had a downward trend (period II: EAPC= -0.04, 95% CI -0.06~-0.02; period III: EAPC= -0.08, 95% CI -0.18~0.00) (Table 3). Decreasing trends in ASPR were observed in both sexes (Fig. 1B). The largest decline in the ASPR from 2011 to 2020 was observed in 2020, with the highest percentage decrease of -30.5% (Table 2). The distribution of ASPRs in regions, sexes and age groups was consistent with the distribution of ASIRs in the same period (Figs. 1B, 2B and 3B).

#### Mortality of PTB

The number of all-cause deaths in PTB patients was 11 234 in Guizhou over 15 years, with an average mortality rate of 2.1 (95% UI: 1.7~2.4) per 100 000 population (Table 2). The number of deaths, annual mortality rate and ASMR had pronounced increasing trends during the past 15 years (Table 2, Fig. 1C). Men had a higher ASMR each year than women (Fig. 1C). The mortality rate of PTB was not detected in all age groups but increased with age and was particularly high in the elderly for both sexes (Fig. 2C). With regard to geographic distribution, the ASMR of PTB remained high in Bijie and Qiannan (Fig. 3C). In 2020, a pronounced increasing trend in ASMR was observed in Anshun (Fig. 3C).

#### DALYs of PTB

The number of DALYs due to PTB was 286 137, with an average DALY rate of 0.60 (95% UI: 0.54~0.66) per 1000 population over the past 15 years in Guizhou (Table 2). The number and rate of DALYs and ASDRs increased during 2006 and 2018 and then decreased slightly after 2019 (Table 2, Fig. 1D). The annual ASDR in males was higher than that in females (Fig. 1D). In both sexes, the DALY rates were unevenly distributed among the age groups. Most cases occurred in elderly individuals, with a peak in those aged 70–79 years (Fig. 2D). In terms of geographic distribution, higher ASDRs were observed in Qiannan and Bijie (Fig. 3D).

### Correlation between GDP per capita and PTB burden

**Fig. 4:**
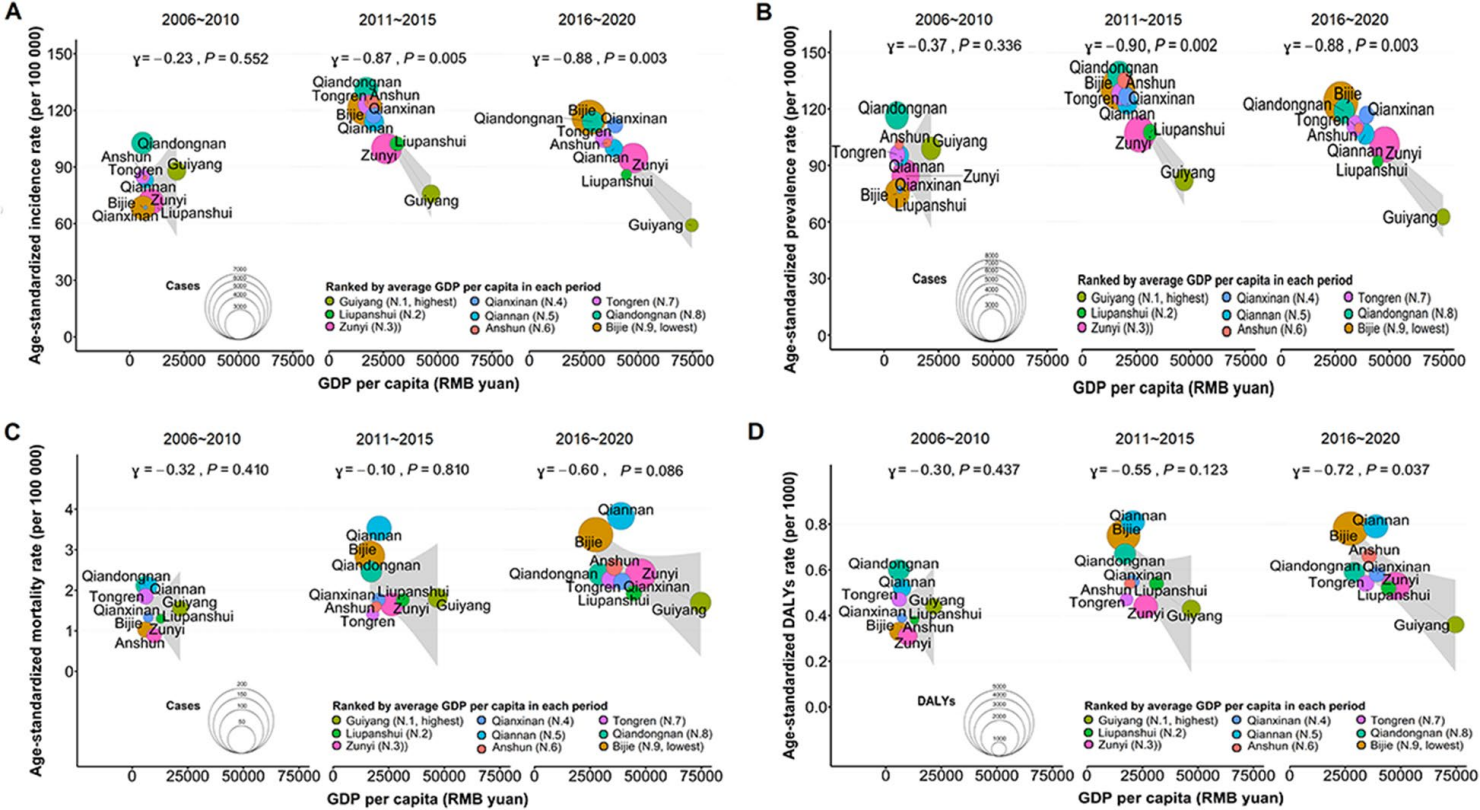
The correlation between GDP per capita and PTB burden in Guizhou Province, 2006-2020. **A** The correlation between average GDP per capita and ASIR of PTB in 3 periods. **B** The correlation between average GDP per capita and ASPR of PTB in 3 periods. **C** The correlation between average GDP per capita and ASMR of PTB in 3 periods. **D** The correlation between average GDP per capita and ASDR of PTB in 3 periods. Notes: PTB: pulmonary tuberculosis; GDP: gross domestic product; ASIR: age-standardized incidence rate; ASPR: age-standardized prevalence rate; ASMR: age-standardized mortality rate; ASDR: age-standardized DALYs rate; DALYs: disability-adjusted life years

According to the level of GDP per capita, 9 prefectures were ranked from highest to lowest: Guiyang (N.1, highest), Liupanshui (N.2), Zunyi (N.3), Qianxinan (N.4), Qiannan (N.5), Anshun (N.6), Tongren (N.7), Qiandongnan (N.8), and Bijie (N.9, lowest). In each period, the GDP per capita of Guiyang was always the highest, while that of Bijie remained the lowest. Figure 4 shows the relationship between GDP per capita and PTB burden for the 9 prefectures in 3 periods. There was a significant negative association between the average GDP per capita and ASIR (2011~2015: ɣ=-0.87, *P*=0.005; 2016~2020: ɣ=-0.88, *P*=0.003), ASPR (2011~2015: ɣ=-0.90, *P*=0.002; 2016~2020: ɣ=-0.88, *P*=0.003) and ASDR (2016~2020: ɣ=-0.72, *P*=0.037). ASMR was not found to have a significant negative correlation with GDP per capita in each period. Thus, further exploration is needed to determine the association between deaths in PTB patients and average GDP per capita over 15 years.

### Risk factors associated with all-cause death and TB specific death

Table 4 shows that a total of 11 234 death cases were reported in Guizhou from 2006 to 2020, including 2233 (19.9%) TB specific deaths and 9001 (80.1%) deaths from other causes. Table 4 also summarizes the results of separate univariate analysis for risk factors associated with all-cause death and TB specific death. Variables with *P* values less than 0.2 after univariate analysis were included in the initial multivariate logistic regression modeling process. Table 5 presents the results from the final separate multivariate logistic regression model. As shown in Table 5, both indicators are found to increase the risk for all-cause death but decrease the risk for TB specific death if the patient is registered in 2 periods (2011~2015 and 2016~2020) or if the patient is in an older age group. Those with a low GDP level were more likely to experience all-cause death but less likely to experience TB specific death. Patients with the following 3 characteristics were more likely to suffer all-cause death and TB specific death: previously treated, no fixed job and receiving DOT during entire treatment. In addition, patients who were male, had severe symptoms or were transferred in were more likely to suffer all-cause death. However, patients who were ethnic minority or found by PCF were more likely to experience TB specific death.

**Table 4:** Univariate analysis for risk factors associated with all-cause death and TB specific death in Guizhou, 2006-2020

Characteristic	Total (n, %)	All-cause death (n, %)			TB specific death (n, %)		
		Yes	No	*P value*	Yes	Death from other causes	*P value*
Total	557476 (100.0)	11234 (2.0%)	546242 (98.0%)		2233 (19.9)	9001 (80.1)	
Three periods				<0.001			<0.001
2006~2010	167421 (30.0)	2390 (21.3)	165031 (30.2)		1063 (47.6)	1327 (14.7)	
2011~2015	203013 (36.4)	3797 (33.8)	199216 (36.5)		838 (37.5)	2959 (32.9)	
2016~2020	187042 (33.6)	5047 (44.9)	181995 (33.3)		332 (14.9)	4715 (52.4)	
Residence ranked by average GDP per capita from 2006 to 2020				<0.001			<0.001
Bijie (N.9, lowest)	105398 (18.9)	2305 (20.5)	103093 (18.9)		307 (13.7)	1998 (22.2)	
Guiyang (N.1, highest)	54220 (9.7)	1163 (10.4)	53057 (9.7)		242 (10.8)	921 (10.2)	
Liupanshui (N.2)	39437 (7.1)	697 (6.2)	38740 (7.1)		233 (10.4)	464 (5.2)	
Zunyi (N.3)	95007 (17.0)	1734 (15.4)	93273 (17.1)		264 (11.8)	1470 (16.3)	
Qianxinan (N.4)	44627 (8.0)	743 (6.6)	43884 (8.0)		173 (7.7)	570 (6.3)	
Qiannan (N.5)	56296 (10.1)	1716 (15.3)	54580 (10.0)		384 (17.2)	1332 (14.8)	
Anshun (N.6)	40548 (7.3)	607 (5.4)	39941 (7.3)		66 (3.0)	541 (6.0)	
Tongren (N.7)	53391 (9.6)	906 (8.1)	52485 (9.6)		304 (13.6)	602 (6.7)	
Qiandongnan (N.8)	68552 (12.3)	1363 (12.1)	67189 (12.3)		260 (11.6)	1103 (12.3)	
Gender				<0.001			0.893
Female	187399 (33.6)	3144 (28.0)	184255 (33.7)		628 (28.1)	2516 (28.0)	
Male	370077 (66.4)	8090 (72.0)	361987 (66.3)		1605 (71.9)	6485 (72.0)	
Age				<0.001			<0.001
≤25	138970 (24.9)	543 (4.8)	138427 (25.3)		185 (8.3)	358 (4.0)	
26-59	285846 (51.3)	4093 (36.4)	281753 (51.6)		944 (42.3)	3149 (35.0)	
≥60	132660 (23.8)	6598 (58.8)	126062 (23.1)		1104 (49.4)	5494 (61.0)	
Ethnic group				0.21			0.055
Han	393276 (70.5)	7865 (70.0)	385411 (70.6)		1601 (71.7)	6264 (69.6)	
Not Han	164200 (29.5)	3369 (30.0)	160831 (29.4)		632 (28.3)	2737 (30.4)	
Occupation				<0.001			<0.001
Fixed job workers and others	61555 (11.0)	476 (4.2)	61079 (11.2)		92 (4.1)	384 (4.3)	
Migrant workers and unemployed	62190 (11.2)	601 (5.3)	61589 (11.3)		166 (7.4)	435 (4.8)	
Farmers	433731 (77.8)	10157 (90.4)	423574 (77.5)		1975 (88.4)	8182 (90.9)	
Classification of patients				<0.001			<0.001
New cases	513805 (92.2)	9895 (88.1)	503910 (92.3)		1915 (85.8)	7980 (88.7)	
Previously treated cases	43671 (7.8)	1339 (11.9)	42332 (7.7)		318 (14.2)	1021 (11.3)	
With severe symptoms				<0.001			0.045
No	488762 (87.7)	9159 (81.5)	479603 (87.8)		1854 (83.0)	7305 (81.2)	
Yes	68714 (12.3)	2075 (18.5)	66639 (12.2)		379 (17.0)	1696 (18.8)	
Types of case finding				<0.001			<0.001
PCF	269144 (48.3)	4855 (43.2)	264289 (48.4)		1150 (51.5)	3705 (41.2)	
ACF	104318 (18.7)	1815 (16.2)	102503 (18.8)		274 (12.3)	1541 (17.1)	
Transfer in	184014 (33.0)	4564 (40.6)	179450 (32.8)		809 (36.2)	3755 (41.7)	
Underwent DOT during whole treatment				<0.001			<0.001
Yes	284005 (50.9)	6137 (54.6)	277868 (50.9)		1302 (58.3)	4835 (53.7)	
No	273471 (49.1)	5097 (45.4)	268374 (49.1)		931 (41.7)	4166 (46.3)	

*TB* Tuberculosis; GDP Gross domestic product, *PCF* Passive case finding, *ACF* Active case finding, *DOT* Directly observed treatment

**Table 5:** Multivariate logistic regression analysis for risk factors associated with all-cause death and TB specific death in Guizhou, 2006-2020

Characteristic	All-cause death			TB specific death		
	Crude OR (95% CI)	Adj. OR (95% CI)	*P* value (LR-test)	Crude OR (95% CI)	Adj. OR (95% CI)	*P* value (LR-test)
Three periods			<0.001			<0.001
Ref: 2006~2010						
2011~2015	1.32 (1.25~1.39)	1.22 (1.16~1.29)		0.35 (0.32~0.40)	0.39 (0.35~0.44)	
2016~2020	1.91 (1.82~2.01)	1.66 (1.58~1.75)		0.09 (0.08~0.10)	0.10 (0.08~0.11)	
Residence ranked by average GDP per capita from 2006 to 2020			<0.001			<0.001
Ref: Bijie (N.9, lowest)						
Guiyang (N.1, highest)	0.91 (0.85~0.97)	0.73 (0.68~0.79)		1.71 (1.42~2.06)	1.41 (1.15~1.73)	
Liupanshui (N.2)	0.80 (0.74~0.88)	0.84 (0.77~0.92)		3.27 (2.68~3.98)	3.27 (2.63~4.06)	
Zunyi (N.3)	0.83 (0.78~0.89)	0.62 (0.58~0.66)		1.17 (0.98~1.40)	1.32 (1.09~1.60)	
Qianxinan (N.4)	0.76 (0.70~0.82)	0.74 (0.68~0.80)		1.98 (1.60~2.43)	1.85 (1.47~2.33)	
Qiannan (N.5)	1.41 (1.32~1.50)	1.03 (0.97~1.10)		1.88 (1.59~2.21)	1.76 (1.47~2.11)	
Anshun (N.6)	0.68 (0.62~0.74)	0.60 (0.55~0.66)		0.79 (0.59~1.05)	0.79 ( 0.58~1.06)	
Tongren (N.7)	0.77 (0.71~0.83)	0.57 ( 0.53~0.62)		3.29 (2.74~3.95)	2.80 ( 2.28~3.44)	
Qiandongnan (N.8)	0.98 (0.91~1.05)	0.91 (0.85~0.98)		1.53 (1.28~1.84)	1.10 (0.90~1.34)	
Gender			<0.001			
Ref: Female				——	——	
Male	1.31 (1.26~1.37)	1.33 (1.28~1.39)		——	——	
Age			<0.001			<0.001
Ref: ≤25						
26-59	3.70 (3.39~4.06)	3.20 (2.91~3.51)		0.58 (0.48~0.70)	0.63 (0.50~0.78)	
≥60	13.34 (12.23~14.58)	11.41 (10.42~12.53)		0.39 (0.32~0.47)	0.53 (0.43~0.65)	
Ethnic group						0.041
Ref: Han	——	——				
Not Han	——	——		0.90 (0.82~1.00)	1.14 (1.01~1.29)	
Occupation			<0.001			0.015
Ref: Fixed job workers and others						
Migrant workers and unemployed	1.25 (1.11~1.41)	0.97 ( 0.86~1.10)		1.59 (1.20~2.13)	1.57 (1.14~2.17)	
Farmers	3.08 (2.81~3.38)	1.49 ( 1.35~1.64)		1.01 (0.80~1.27)	1.21 (0.94~1.58)	
Classification of patients			<0.001			0.022
Ref: New cases						
Previously treated cases	1.61 (1.52~1.71)	1.46 (1.38~1.55)		1.30 (1.13~1.48)	1.20 (1.03~1.39)	
With severe symptoms			<0.001			0.128
Ref: No						
Yes	1.63 (1.55~1.71)	1.47 (1.40~1.54)		0.88 (0.78~0.99)	0.90 (0.78~1.03)	
Types of case finding			<0.001			<0.001
Ref: PCF						
ACF	0.96 (0.91~1.02)	0.99 (0.94~1.05)		0.57 (0.50~0.66)	0.70 ( 0.59~0.81)	
Transfer in	1.38 (1.33~1.44)	1.21 (1.16~1.26)		0.69 (0.63~0.77)	0.93 (0.83~1.04)	
Underwent DOT during whole treatment			<0.001			<0.001
Ref: No						
Yes	1.16 (1.12~1.21)	1.08 (1.04~1.12)		1.20 (1.10~1.32)	1.23 (1.11~1.37)	

*TB* Tuberculosis, *Adj.* Adjusted, *OR* Odds ratio, *CI* Confidence intervals, *IR-test* Likelihood ratio test, *GDP* Gross domestic product, *PCF* Passive case finding, *ACF* Active case finding, *DOT* Directly observed treatment

## Discussion

This study collected a large sample size of 557 476 PTB cases and 11 234 deaths from the TBIMS database over 15 years (2006-2020) in Guizhou Province. This is the first comprehensive assessment of the PTB burden and risk factors for all-cause death and TB specific death in this province to advance evidence-informed prevention plans. Our main finding shows that there was a downward trend in TB specific deaths and the incidence and prevalence of PTB. However, all-cause mortality and DALYs had an upward trend in the past 15 years driven by deaths from other causes. The highest PTB burden was observed among males, people aged 60 and above, and in 5 prefectures with relatively low economic levels. Patients with no fixed job, those previously treated and those receiving DOT were more likely to suffer all-cause death and TB specific death. Patients registered from 2011 to 2020 and those with older age increased the risk for all-cause death but decreased the risk for TB specific death. Those with a low GDP level were more likely to experience all-cause death but less likely to experience TB specific death. Patients who were male, had severe symptoms or were transferred were more likely to suffer all-cause death. However, patients who were ethnic minority or found by PCF were more likely to experience TB specific death.

Our study shows that the highest PTB caseloads were among young to middle-aged adults, and more than nine out of ten were new PTB cases. These results concur with previous studies conducted in China [7, 42]. The proportion of new patients, female patients, minority patients and elderly patients has increased over time, which was probably due to the good performance of the ACF strategy implemented in China since 2011 [4, 5]. ACF plays an important role in TB case finding, especially in students [43], elderly individuals [44] and contacts [35]. However, only half of the patients in our study underwent DOT throughout the duration of treatment, and this proportion fell to less than 50% between 2016 and 2020. DOT is the core component of DOTS [45]. The proportion of all PTB patients administered DOT is one of the key indicators for assessing the level of TB care and prevention [36]. Inadequate implementation of DOT leads to poor treatment adherence and further generates the emergence of TB drug resistance [45]. Thus, our findings suggest that incentives and supervision mechanisms should be established to encourage DOT providers to manage cases effectively and to assess their performance regularly.

In this study, temporal trends of ASRs show that the highest PTB burden was observed in 2008. This may be due to the adequate case detection and notifications after full coverage of DOTS and TBIMS during the period from 2006 to 2010 [46]. Since 2012, both the overall ASIR and ASPR of PTB showed a downward trend in Guizhou, which was consistent with the trends of PTB nationwide [47]. Meanwhile, the number in TB specific deaths consistently decreased during the study period in Guizhou. These decreases reflect the effectiveness of TB control measures in recent decades. A sharp decline was observed in the ASIR and ASPR in 2020. One of the possible reasons may be attributed to the impact of the COVID-19 pandemic on TB services [1]. Globally, the number of TB patients newly diagnosed and reported had a large drop in 2020 compared with 2019 due to COVID-19 [1]. Another possible reason might be explained by some effectiveness of wearing face masks in the community to prevent respiratory disease infection risk [48]. However, more reasons are worth further exploration to guide appropriate policy decisions during high-risk periods. Additionally, it is important to highlight that PTB mortality and DALYs had an upward trend in the past 15 years in Guizhou, primarily due to the continuous increase in deaths from other causes over the same period. Death from other causes in PTB patients might be caused by adverse drug effects [49] and common comorbidities, such as TB-DM (diabetes mellitus) [50], TB-HIV positivity [51] and other age-associated comorbidities [49]. Therefore, our findings suggest that reducing the TB disease burden in Guizhou will require a multipronged approach, including health system strengthening, integration of TB and common comorbidity services, and person-centered approaches to support treatment.

Consistent with previous studies [18, 42], a gender disparity in PTB burden was observed in our study, with males being more affected in all age groups. This can be explained by smoking [16, 42], more frequent exposure to TB bacillus in work activities, and lower levels of self-care and health-care use for TB in this gender [16, 19]. Adult males comprise the main labor resources. Loss of their jobs or reduced productivity due to TB could generate a negative socioeconomic impact for them, their family and society [16]. Furthermore, our study found that the PTB burden increased with age and mainly fell on people aged 60 and above. The elderly population faces many challenges that make them more susceptible to PTB infection and unfavorable treatment outcomes [42]. The challenges include decreased lung function, low tolerance to anti-TB drugs and increased comorbidity [49]. These results suggest that more attention should be given to males and elderly people. The expansion of financial and social protection could improve TB prevention and increase favorable TB treatment outcomes among vulnerable populations [16].

Regional variations in the PTB burden were observed in this study, and they were mainly concentrated in 5 prefectures (Bijie, Anshun, Tongren, Qiandongnan and Qiannan) with low economic levels in Guizhou. Our findings show a clear negative association between the PTB burden and GDP per capita of a prefecture, which corroborates previous findings [1, 52]. The following reasons might explain this phenomenon. First, areas with lower economic development have insufficient spending on financial and social protection, public health expenditure and hospital funding for TB. Second, areas with underdeveloped economies have weaker health systems and a lack of adequate health services and trained human resources. Third, people living in economically underdeveloped areas have relatively weak educational levels and health-care awareness. They will use less income for health care and will not see a doctor unless there is a major illness. TB is fundamentally a disease of poverty [53]. Thus, the WHO End TB Strategy highlights the importance of socioeconomic determinants for TB prevention and treatment [52–54]. Both economic development and increasing social protection spending can reduce the TB burden [52].

In terms of risk factors associated with deaths in PTB patients, first, our study shows that patients with no fixed job, those previously treated and those receiving DOT were more likely to suffer all-cause death and TB specific death. Similar results have been reported in other studies. Low income [29] and illiterate schooling [32], previous TB treatment [30, 31, 51]and the use of DOT during the entire treatment [55] were independently associated with death in TB patients. A possible explanation for the correlation between DOT and death was that DOT was utilized by providers according to decreased social support or increased severity of illness [55]. However, additional studies are needed to evaluate the quality of DOT implementation in the treatment of TB patients in Guizhou. Second, our findings show that patients registered from 2011 to 2020, those with older age and those with severe symptoms were more likely to experience all-cause death but less likely to experience TB specific death. In our study, of the all-cause deaths, 19.9% were TB specific deaths, and 80.1% were deaths from other causes. The number in TB specific deaths decreased from 2011 to 2020, reflecting the considerable success of Guizhou's anti-TB program. Local governments and political organizations have taken action to control TB through improved TB health care systems and financial initiatives [8, 11]. However, the upward trend in deaths from other causes means that TB control in Guizhou still faces many challenges, such as the emergence of drug resistance, more individuals with TB-DM and other age-related comorbidities. Third, this study found that patients who were male or those who were transferred in were more likely to suffer all-cause death. These results correspond with the findings from other studies. Male TB patients with low treatment adherence, smoking, alcohol or drug use are associated with high death [32, 56]. The process of patient referral leads to a delay in diagnosis and treatment, which increases mortality rates among PTB patients [30, 57]. Finally, patients who were ethnic minority or found by PCF were more likely to experience TB specific death. These factors may be explained by ethnic groups with TB living away from health facilities; PCF delays their seeking TB care and leads to unsuccessful treatment outcomes [35, 58]. Thus, ACF is beneficial for vulnerable people to overcome barriers to accessing health services [35, 58]. Therefore, our findings suggest that targeted interventions are needed to address these risk factors to reduce deaths among TB patients in Guizhou.

This study has several limitations. First, only PTB patients based on the TBIMS database were included in this study. As PTB accounts for more than 90% of TB patients in China [7], our results could represent the overall epidemic of TB in Guizhou. Second, underreporting of TB cases and missing data were inevitable when using notification data collected from the existing surveillance system [7, 59, 60]. In our study, after 348 patients are excluded, the remaining 557,476 cases are still representative of the TB status for this province. Third, the data from the surveillance system only contain limited variables. Some risk factors for the TB burden in the GBD were not available in the TBIMS database, such as smoking [7, 15–17], alcohol use [7, 15–17], diabetes [15, 16] and sociodemographic index (SDI) [15]. Thus, their effects on PTB burden could not be assessed in this study. Meanwhile, we could also not assess those potential variables associated with deaths among TB patients, such as “bacterial number” and drug-resistant status of patients, treatment regimen, comorbidities and complications. Finally, our analysis of the relationship between GDP per capita and PTB burden (ASIR, ASPR, ASMR and ASDR) cannot be interpreted as causal, as it reflects only the average historical correlation between GDP per capita and each of the measures [15, 19, 20]. Despite these limitations, the results of this study should be useful for policy decisions and spur future studies to resolve the limitations and obtain more precise results.

## Conclusions

This study systematically estimated the PTB burden and risk factors for deaths among PTB patients from 2006 to 2020 for the first time in Guizhou. There was a downward trend in TB specific deaths and the incidence and prevalence of PTB. However, all-cause mortality and DALYs increased due to deaths from other causes. Gender and age disparities showed that the burden of PTB was higher in men and elderly individuals. Regional heterogeneity revealed that the PTB burden was stably clustered in Bijie, Anshun, Tongren, Qiandongnan and Qiannan, which had low economic levels. Patients with no fixed job, those previously treated and those receiving DOT were more likely to suffer all-cause death and TB specific death. Patients registered from 2011 to 2020, those with older age, those with severe symptoms, and those with a low GDP level were more likely to experience all-cause death but less likely to experience TB specific death. Patients being male or those transferred in significantly increased the likelihood of all-cause death. Being an ethnic minority or PCF was associated with TB specific death. Our findings suggest that a multipronged and targeted approach is needed in Guizhou to strengthen the healthcare system, address modifiable risk factors and increase healthcare in high-risk PTB groups and areas. Reduction in the TB burden in Guizhou will diminish the overall burden of TB in China and will be crucial in reaching the targets of the End TB Strategy.

## Data Availability

The datasets generated and analyzed during the current study are not publicly available due to the fact that it contains personal information, but are available from the corresponding author on reasonable request.

## Abbreviations

TB: Tuberculosis
PTB: Pulmonary tuberculosis
COVID-19: Coronavirus disease 2019
DOTS: Directly observed treatment short-course strategy
WHO: World Health Organization
TBIMS: TB Information Management System
GBD: Global burden of diseases
DOT: Directly observed treatment
GDP: Gross domestic product
PCF: Passive case finding
ACF: Active case finding
IQR: Interquartile ranges
DALYs: Disability-adjusted life years
ASRs: Age-standardized rates
ASIR: Age-standardized incidence rate
ASPR: Age-standardized prevalence rate
ASMR: Age-standardized mortality rate
ASDR: Age-standardized DALYs rate
UIs: Uncertainty intervals
Loess: Locally weighted regression
EAPCs: Estimated annual percentage changes
CI: Confidence interval
OR: ODDS ratio
SDI: Socio-demographic Index
